# Communication Intervention to Improve Young Adults’ Food Safety Practices: The Benefits of Using Congruent Framing

**DOI:** 10.3390/nu17050928

**Published:** 2025-03-06

**Authors:** Michela Vezzoli, Valentina Carfora, Patrizia Catellani

**Affiliations:** 1 DISTUM, Department of Humanities, University of Urbino Carlo Bo, 61029 Urbino, Italy; 2 Department of Psychology, Catholic University of the Sacred Heart, 20123 Milan, Italy; 3 Faculty of Economics, International University of Rome, 00147 Rome, Italy; valentina.carfora@unint.eu (V.C.);

**Keywords:** mHealth intervention, food safety, young adults, domestic environment, framing, frame congruency, behaviour change

## Abstract

**Background/Objectives:** Improving food safety practices among young adults is critical to public health, but effective communication strategies are under-researched. This study investigated the effectiveness of a 12-day message-based intervention to promote safe food handling practices using a randomised controlled trial. **Methods:** A total of 588 participants (aged 18 to 35 years) were randomly assigned to one of four experimental conditions or to a control group. Participants in the intervention groups received daily messages via a mobile app, while the control group received no messages. The intervention combined belief-based content to raise awareness with skill-based content to teach practical food handling, framed by either positive or negative emotional appeals. The experimental conditions differed in message congruence, with belief-based and skill-based content framed either consistently (both positive or both negative) or inconsistently (one positive, one negative). To assess the impact of the intervention, self-reported adherence to food safety practices, food safety awareness, and self-efficacy were measured at baseline and post-intervention. **Results:** The results showed that the intervention significantly improved food safety practices, especially when the messages were congruent in valence. Pre-intervention self-efficacy moderated the effects, with higher self-efficacy increasing receptivity to certain messages, while lower self-efficacy benefited from a different framing. Self-efficacy, but not awareness, mediated behaviour change, highlighting its key role in the success of the intervention. **Conclusions:** These results emphasise the importance of message valence congruence and individual self-efficacy levels in designing effective food safety interventions. Future research should investigate long-term intervention effects, adaptive mHealth strategies, and tailored communication approaches to maximise engagement and sustained behaviour change.

## 1. Introduction

Food safety involves the handling, preparation, and storage of food in a manner that minimises the risk of foodborne illness [1]. Each year, millions in Europe suffer from foodborne illnesses, with significant public health, economic, and social consequences [2,3]. As the final link in the food safety chain, consumers play a critical role in preventing foodborne illness through responsible food handling, preparation, and storage [4,5,6]. The home environment is a major source of foodborne disease, where poor hygiene, inadequate food handling, and improper temperature control create conditions for microbial growth [7,8,9]. Young adults (18–35 years) are particularly prone to unsafe food practices due to limited experience and knowledge, often stemming from insufficient exposure to food safety principles [10,11,12,13]. While not traditionally considered at-risk, their food handling behaviours are critical as they transition into roles as caregivers or food service workers. Educating young adults on food safety is essential for fostering consistent safe practices at home and beyond, promoting a culture of food safety compliance [14].

Most interventions to improve food safety fall into two main categories or combine elements of both: (1) training workshops, courses, and curricula offered in schools, universities, and communities, and (2) social marketing campaigns and educational materials, including print media (e.g., brochures, websites, information on food labels) and audiovisual media (e.g., radio and television commercials) [15,16,17,18]. Food safety interventions have been assessed using a variety of measures, ranging from behavioural and psychological factors (e.g., knowledge, attitudes, intentions, perceived risk) to objective indicators of microbial contamination. These interventions can significantly improve behaviour, with effects ranging from 10% to 65% depending on the type of the intervention. Workshops and hands-on training had the greatest impact, with up to 65% of participants improving their behaviour (e.g., hand washing, proper food storage), while short-term awareness campaigns had a limited lasting impact, as behavioural improvements often faded after 3–6 months if not reinforced [15,16,17,18].

Despite their effectiveness, these interventions face significant limitations. High costs for materials, trainers, and venues make large-scale implementation challenging, while substantial time commitments and logistical difficulties, such as scheduling and attendance, further hinder their effectiveness. In this context, mHealth applications (i.e., the use of mobile and wireless devices to improve health) present a scalable and cost-effective alternative [19]. These tools are accessible anytime and anywhere, offering convenience for users and minimal additional costs for distribution. There is evidence of the effectiveness of messages and digital interventions in promoting food safety behaviours. They show a moderate impact, with behavioural improvements ranging from 10% to 35%, depending on message framing and reinforcement strategies [17]. These results highlight the flexibility and scalability of such interventions, making them valuable tools for targeted, wide-ranging food safety education [19,20]. This adaptability makes mHealth a valuable tool in various health areas, allowing interventions to be tailored to specific behaviours and needs. However, its potential to promote safe food practices among young adults at home remains unexplored, offering an opportunity to empower this group to adopt crucial food safety habits.

To address this problem, in this study, we tested a 12-day intervention aimed at young adults and delivered via the smartphone app PsyMe. The PsyMe app (https://centridiricerca.unicatt.it/psylab-home-psyme-app) is a free smartphone application designed to support scientific research in social psychology and artificial intelligence. To ensure participants’ privacy and anonymity, the app assigns a unique anonymous code to each user. PsyMe facilitates the distribution of questionnaires, messages, and push notifications, making it easy to remind participants to engage with the content. Our intervention aimed to improve safe food handling and storage at home by increasing young adults’ beliefs about the impact of their actions (i.e., food safety awareness) and their confidence in carrying out these practices (i.e., self-efficacy). To achieve this goal, our communication manipulated both the content and the framing of the message. In terms of message content, we combined *belief-based content* that emphasised the importance of safe food practices with *skill-based content* that provided practical guidance on safe food practices. In designing the messages, we examined the valence congruence of the messages and explored the potential differential effects of *congruent valence* (i.e., when both message contents have the same positive or negative framing) and *incongruent valence* (i.e., when one content is framed positively and the other negatively, or vice versa). Since the effectiveness of messages can vary depending on the psychological characteristics of the recipients [21], this study also investigated how our messages were received by individuals depending on their awareness of and self-efficacy towards food safety practices. This research could contribute to the development of a tailored approach to promote lasting changes in young adults’ food safety behaviours.

### 1.1. Theoretical Background

To develop effective communication to promote food safety, it is important to address not only what content should be presented but also how to frame this content to maximise its impact and how both features can be differently effective depending on the characteristics of the recipients. Furthermore, it is crucial to consider how different approaches may vary in their effectiveness depending on characteristics of the recipients [22].

#### 1.1.1. Message Content: Leveraging Beliefs and Skills Related to Food Safety Practices

Food safety awareness refers to the knowledge and understanding of practices, behaviours, and risks related to preventing foodborne illness and ensuring safe food handling throughout the supply chain, from production to consumption [23]. Experimental studies suggest that raising awareness is an effective strategy for promoting food safety practices. For example, Karg and Drechsel [24] found that raising food safety awareness among farm workers significantly increased the adoption of preventive measures across the food production chain. However, awareness alone is not always enough to change behaviour. A meta-analysis of food safety interventions found that while awareness improved, actual food handling behaviours often remained unchanged [17]. Consequently, a holistic approach that tailors interventions to specific behaviours and contexts is recommended for more effective food safety education [25].

Another strategy to promote food safety is to strengthen self-efficacy [26], which refers to an individual’s belief in their ability to perform behaviours necessary to achieve desired outcomes [27]. Self-efficacy reflects confidence in one’s ability to consistently apply safe food handling practices, even in challenging or resource-limited environments [28,29]. High self-efficacy bridges the gap between knowledge and action, enabling individuals to overcome barriers such as limited resources or competing priorities and translate knowledge into action [14,30]. Previous research has shown that interventions, including goal-setting and hands-on training, are effective in increasing self-efficacy and improving food safety behaviours [26,28]. Studies show that interventions combining knowledge-building with self-efficacy enhancement yield superior outcomes [28]. For example, Kim et al. [31] demonstrated that their educational program incorporating lessons, quizzes, and discussions effectively strengthened adolescents’ self-efficacy and food safety practices. Likewise, Wong et al. [32] found that a 2 h on-site training, followed by a 14-day self-efficacy intervention, significantly improved compliance, knowledge, and self-efficacy among school canteen workers.

Based on the above findings, in this study, we integrated *belief-based* and *skill-based content* to influence individuals’ food safety awareness and increase self-efficacy. To design the belief-based content, we used a prefactual language style, which is an effective method for influencing beliefs, by presenting hypothetical scenarios in an “if... then” format [33] (e.g., “If you keep your refrigerator at 4 °C, then your food will stay safe”). This technique effectively drives behaviour change by prompting individuals to mentally simulate the positive or negative consequences of their actions. It has proven effective in shaping perceptions and behaviours related to healthy and sustainable dietary choices [34,35]. Meanwhile, we conceptualised skill-based content as “how-to” suggestions that provide precise guidance on the specific actions individuals should perform, how they should perform them, and when they are most effective [36]. By providing clear, step-by-step instructions, this approach breaks down complex behaviours into manageable tasks and makes behaviour change more accessible. Research demonstrates that explicit, detailed instructions not only increase individuals’ understanding but also increase their confidence and sense of ability to implement safe food practices [37].

Based on the above literature, in the present study, we hypothesised that participants exposed to food safety messages (with a combination of belief- and skill-based content) would show an increase in food safety awareness (Hypothesis 1a; H1a), perceived self-efficacy (Hypothesis 1b; H1b), and self-reported food safety practices (Hypothesis H1c; H1c) compared to participants in the control condition.

#### 1.1.2. Message Framing: Comparing Congruent and Incongruent Valence

According to Framing Theory [38], individuals interpret messages differently depending on the perspective or “frame” from which the information is presented. The framing effect suggests that people are not always rational decision-makers and can be influenced by the cues in a message’s framing [39]. Research has found that the persuasiveness of messages in behaviour change contexts often depends on whether the framing of the message has a positive or negative valence [40,41]. In this context, *valence* refers to whether the message emphasises the gains of performing the recommended behaviour (*positive valence*) or highlights the losses of failing to perform the behaviour (*negative valence*). Previous findings on preventive health-related actions (i.e., actions that are considered very low risk except that the greatest risk is not performing them, such as exercising or using sunscreens [42]) and low-risk behaviours suggest that positively framed messages are often more effective than negatively framed messages [43,44,45]. However, meta-analyses [40,46] suggest that while positively framed messages offer some benefits, their effectiveness is modest and not always applicable across all contexts.

Categorising food safety behaviours is not straightforward. While preventative, they are not always perceived as low risk, as failing to follow proper practices can have serious consequences. As a result, it remains unclear whether positive or negative valence is more effective in promoting compliance. Additionally, no studies have specifically examined the impact of message valence alignment on food safety promotion. This study addresses this gap by exploring valence congruence between belief-based and skill-based content, either matching or contrasting their positive or negative valence. Research suggests that congruent messages—where both components share the same valence—are more effective at influencing behaviour than incongruent ones. For example, studies on image–text valence alignment show that matching emotional tone enhances persuasion. Chang and Lee [47] found that advertisements were more successful in encouraging charitable donations when the image and message valence aligned (e.g., negative image with a negatively framed message). Similarly, Lee and Cho [48] demonstrated that pro-environmental ads were more persuasive when the message and image framing matched, leading to greater attitude shifts and purchase intentions. In the context of physical activity, Howlett and colleagues [49] showed that positively framed messages paired with positive images increased intentions and self-efficacy more effectively than incongruent ones.

Few studies have explored framing congruence in text messages. Ngo and colleagues [50] examined congruence in construal level (information- vs. action-oriented content) rather than valence, finding that aligned messages—whether abstract/informational or concrete/action-oriented—were more effective in shaping behavioural intentions than incongruent ones. In terms of valence congruence, two studies suggest that the inclusion of mixed valences in a message may be more beneficial. Mukherjee & Dubé [51] found that combining humour with fear appeals reduced defensive reactions, increasing persuasiveness. This supports the idea that mixing positive and negative emotions cushions overarousal and enhances information processing, as positive emotions counterbalance negative intensity [52]. Similarly, research by Rossiter and Thornton [53] showed that fear appeals ending on a positive note—referred to as “happy endings”—reduced defensive reactions and improved compliance. In addition, Siegenthaler and colleagues [52] found that fear appeals about work-related stress were more effective when they transitioned from a threat to positively framed efficacy information. This positive valence shift improved efficacy perceptions, particularly among those who found the health issue personally relevant or experienced higher stress levels in their daily lives.

These findings suggest that while valence congruence (matching positive or negative tones) can enhance persuasion, a mixed-valence approach may reduce defensiveness and boost engagement. Accordingly, in our study, we investigated whether aligning or contrasting belief-based and skill-based content in terms of valence improves message effectiveness in a first attempt to understand the role of valence congruence in food safety communication. Specifically, we addressed the following research questions: Does belief-based and skill-based content congruence or incongruence affect food safety awareness (Research Question 1a; RQ1a), perceived self-efficacy (Research Question 1b; RQ1b), and self-reported practices (Research Question 1c; RQ1c)?

### 1.2. The Moderating Role of Food Safety Awareness and Self-Efficacy

Communication research has shown that the effectiveness of appeals depends on a considerable extent on the alignment between the framing of a message and the characteristics of the person or context [21,54,55]. This suggests that message-based interventions vary greatly in effectiveness from person to person, with a key factor being the baseline characteristics of the recipients (i.e., pre-existing characteristics prior to the intervention). Baseline characteristics have a significant impact on whether and how well a person responds to a particular intervention. For example, in food safety communication, Bai and colleagues [56] found that individuals with higher levels of personal involvement (e.g., health concerns or previous food safety incidents) showed greater acceptance of both gain and loss messages than those with lower levels of involvement. Similarly, Jin and Han [57] observed that consumers’ responses to food safety messages depended on subjective prior knowledge, with those who knew less about food safety issues being more influenced by the message framing than those with higher levels of knowledge. Our study builds on this line of research by examining whether the effectiveness of communication interventions to promote food safety practices is moderated by baseline food safety awareness and self-efficacy.

Baseline food safety awareness and self-efficacy can significantly influence the effectiveness of interventions to improve food safety behaviours. Chow and Mullan [58] found that when individuals recognise their susceptibility to foodborne illness, understand the benefits of proper food handling, and believe they can overcome potential barriers to safe behaviours, the intention to prepare food hygienically develops. While some studies have shown that awareness moderates the effect of various psychosocial predictors, such as attitude and health consciousness on intentions to make healthy food choices [59], no study to date has examined whether pre-existing beliefs about the consequences of behaviour (specifically, awareness that improper food handling poses significant health risks) moderate the effectiveness of such messages on people’s actual behaviour. This gap in the literature highlights the need to explore how differences in food safety awareness affect the effectiveness of communication strategies to promote safe food practices.

Regarding the latter, previous research shows that individuals with high self-efficacy are strongly influenced by health messages, especially those that are negatively framed [60,61], as they feel confident that they can act and avoid negative consequences. Conversely, individuals with low self-efficacy tend to respond less to messages [62,63], with mixed results on whether they are more influenced by positive or negative framing [34]. However, despite evidence from broader contexts, no research to date has specifically examined the role of self-efficacy in moderating the effectiveness of food safety behaviour improvement interventions. This gap highlights the need to examine how differences in self-efficacy may impact the success of food safety communication strategies.

With this in mind, in this study, we examined how food safety interventions can promote safe practices through its effects on awareness and self-efficacy, while also examining whether baseline levels of these traits moderate intervention outcomes. Therefore, we investigated the following research question:

Do pre-intervention food safety awareness and self-efficacy moderate the effectiveness of the message-based intervention to increase adherence to safe food practices? (RQ2).

Do pre-intervention food safety awareness and self-efficacy moderate the mediating pathway linking the intervention to adherence to safe practices through improved awareness and self-efficacy? (RQ3).

## 2. Materials and Methods

### 2.1. Sample

We estimated the sample size using GPower 3.1 [64]. A sample of 520 participants is considered sufficient to detect a small-to-medium effect size (f = 0.15; [65]), with 90% power and α = 0.05 in a factorial ANOVA with five between-subject manipulated conditions and two repeated measures.

Italian young adults were invited to participate in the study as volunteers by the students of the Catholic University of the Sacred Heart. Participants received no incentives for participation. To be eligible for the study, participants had to be between 18 and 35 years old and cook for themselves and/or others more than twice per week. Previous studies used cooking at least twice a week as a threshold to distinguish people who cook frequently from those who cook infrequently [66]. Of the 1267 individuals who completed the screening questionnaire, 1065 met the eligibility criteria. However, 477 participants were excluded for various reasons: incomplete questionnaires (Time 1: *n* = 35; Time 2: *n* = 213), technical problems that prevented delivery of the messages (*n* = 127), failure to pass the attention check questions (*n* = 26), and low engagement, defined as reading fewer than 5 out of 12 messages (*n* = 76). This resulted in a final sample of 588 participants: 332 participants identified as female (56%), 254 as male (43%), 1 person identified as non-binary (0.2%), and 1 participant chose not to provide a response (0.2%). The participants had an average age of 23.53 years (SD = 3.68), and 73% (*n* = 423) of them were either currently pursuing or already obtained a bachelor’s or master’s degree.

**Figure 1:**
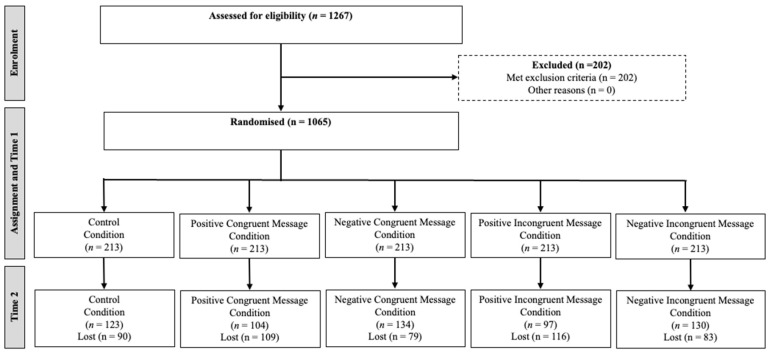
Flow chart of participants through each stage.

Figure 1 shows the flow chart of the participants during the different phases of the study.

### 2.2. Design and Procedure

This study was conducted with the approval and supervision of the Ethic Commission of the Catholic University of the Sacred Heart (Milan). To collect the data, we asked 80 psychology students to invite three Italian women and three Italian men between the ages of 18 and 35 to participate in the study via e-mail or text message. Each participant first provided their consent to participate in the study and completed a screening questionnaire to confirm eligibility. This assessed age and frequency of food preparation at home against the study inclusion criteria. Eligible participants then completed the first questionnaire (Time 1), which included self-report measures of safe food-related behaviours, food safety awareness, and self-efficacy. This questionnaire took approximately 12 min to complete.

Following the questionnaire at Time 1, participants were randomly assigned to one of four message conditions or the control condition (see *Message Intervention* section). Only participants in the four message conditions received daily messages for a period of 12 days. At the end of this intervention period, all participants completed a follow-up questionnaire (Time 2), which looked like Time 1 but contained additional questions specifically related to the intervention. These additional questions assessed perception of the messages (e.g., perceived tone of voice), engagement with the messages (e.g., involvement, trust in the information, depth of processing), and emotional responses (e.g., perceived threat to freedom, elicited emotions). Participants in the control condition did not receive or rate any messages. This questionnaire took approximately 15 min to complete.

### 2.3. Measures at Time 1

The questionnaire at Time 1 included several measures, all of which are available on OSF. Here, we report the measures relevant to this study, i.e., food safety practices, awareness, and self-efficacy. The adapted scales were all translated into Italian and, specifically for awareness and self-efficacy, adapted to the food safety context.

*Adherence to food safety practices (adapted from [8]).* We measured how frequently respondents practiced safe food handling, with focus on meal preparation and storage behaviours. Respondents rated 20 items (e.g., “I clean work surfaces with a cleaning agent when I finish preparing a meal”, “I only put food in the freezer when it is not hot”) on a 5-point Likert scale (1 = Never; 5 = Always). Higher scores correspond to greater adherence to food safety practices (α_Time1_ = 0.709, ω_Time1_ = 0.739; α_Time2_ = 0.801, ω_Time2_ = 0.827).

*Food safety awareness (adapted from [67]).* We assessed individuals’ knowledge and perceptions of the health risks associated with improper food handling. Participants rated three items (i.e., “Improper food preparation is one of the causes of health problems”, “Improper food storage is one of the causes of health problems”, and “More information about proper food preparation and storage would help to protect health”; adapted from [66]) on a 7-point Likert-type agreement scale (1 = Completely Disagree; 7 = Completely Agree). Higher scores correspond to greater food safety awareness (α_Time1_ = 0.669, ω_Time1_ = 0.679; α_Time2_ = 0.801, ω_Time2_ = 0.808).

*Self-efficacy (adapted from [68]).* We assessed participants’ confidence in their ability to handle and store food safely despite emotional, social, and situational barriers. Participants rated 12 items (e.g., “when you feel anxious or bored?”; “when you have to prepare many meals”) on a 7-point Likert scale about how confident they were that they could perform it anyway (1 = Not at all confident that I can do it; 7 = Completely confident that I can do it). Higher scores correspond to greater confidence in performing safe food handling behaviour (α_Time1_ = 0.900, ω_Time1_ = 0.901; α_Time2_ = 0.922, ω_Time2_ = 0.922).

### 2.4. Message Intervention

Each participant was randomly assigned to one of five conditions. Participants in the experimental conditions received an introductory message defining food safety and eleven condition-specific messages. The messages were sent daily for 12 days via the PsyMe app. The eleven food safety messages were developed based on a selection of categories of food-related behaviours, identified using the Food Preparation and Storage Scale [8]. These categories included essential practices to ensure food safety and minimise health risks. In particular, these included proper refrigeration (1), proper storage of newly purchased food (2), safe storage of leftovers (3), safe freezing methods in terms of duration (4) and temperature (5), proper thawing techniques (6), attention to water quality (7), proper use of food packaging and containers (8), hand hygiene during food preparation (9), cleanliness of utensils and the preparation environment (10), and compliance with cooking times and temperatures (11). These categories were selected through a peer review process involving experts in the field of food safety.

**Figure 2:**
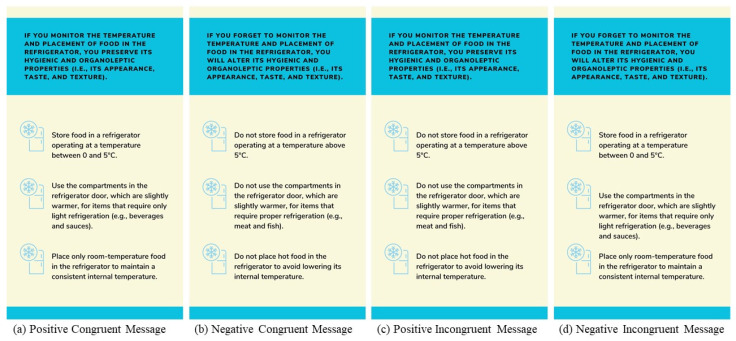
Examples of congruent or incongruent messages framed positively or negatively.

Each condition-specific message contained a belief-based content (a prefactual statement describing the possible consequences of food-related behaviours) and a skill-based content (two to four practical instructions). Both contents were manipulated to reflect either congruent or incongruent valence. This gave us four message conditions. Figure 2 shows an example of the messages for each condition (the full list of messages can be found on OSF). The *positive congruent condition* (PC condition; *n* = 130) included both positively framed belief- and skill-based contents. The *negative congruent condition* (NC condition; *n* = 104) included both belief- and skill-based contents that were negatively framed. The *positive incongruent condition* (PI condition; n = 97) contained positively framed belief-based content and negatively framed skill-based content. The *negative incongruent condition* (NI condition; *n* = 134) contained negatively framed belief-based content and positively framed skill-based content. Participants in the control condition (*n* = 123) did not receive any messages during the intervention phase, and participants were evenly distributed across conditions, χ^2^(4) = 9.024, *p* = 0.061.

The introductory message was also framed to match the participant’s assigned condition. Participants in the PC and PI conditions received a positively framed introductory message emphasising the health benefits of safe food practices. In contrast, under the NC and NI message conditions, participants received the introductory message in a negative frame that focused on the risks to health and safety from improper food handling (“Dear Participant, food safety is very important to avoid health risks. It is about adopting a set of habits to ensure that food is handled and consumed at home in a way that avoids risks to health and hygiene”). Then, all participants were told the following: “Over the next few days, we will be sending you some messages about safe food preparation and storage. Please read each message carefully. You will receive a notification when you can read a new message”. All messages were sent in the morning. Those who had not read the message by the afternoon received a reminder.

### 2.5. Measures at Time 2

In the questionnaire at Time 2, we again measured food safety awareness, self-efficacy, and behaviour using the same measures as at Time 1. We also included some scales to assess participants’ evaluations of the messages received via the PsyMe app. Participants in the control group did not answer these scales.

*Frequency of message reading.* Participants were asked to indicate how often they read the messages they received by rating a single item on a scale from “never” (1) to “always” (5).

*Message tone.* The perceived tone of the messages was measured with a single item (“Overall, how would you rate the tone of the information presented in the messages you read?”) on a semantic difference scale ranging from “extremely negative” (1) to “extremely positive” (7). Higher scores indicated a more positive perception of the information tone.

*Message involvement (as used in [69]).* This scale measured the extent to which the messages engaged participants cognitively and affectively with three items (e.g., “The messages involved me in what they had to say”) rated on a 7-point Likert scale (1 = Completely disagree; 7 = Completely agree). Higher scores correspond to greater engagement (α = 0.832; ω = 0.835).

*Trust in the messages (as used in [69]).* This scale assessed the perceived credibility, reliability, and truthfulness of the information presented in the messages with three items (e.g., “How much trust do you have in the information presented?) rated on a 7-point Likert scale (1 = Completely disagree; 7 = Completely agree). Higher scores correspond to greater trust in the messages (α = 0.908; ω = 0.911).

*Systematic processing (as used in [69]).* This scale assessed the depth and thoroughness with which participants engaged with and thought about the information presented in the messages with five items (e.g., “As I read the messages, I thought about what actions I might take based on what I read”) rated on a 7-point Likert scale (1 = Completely disagree; 7 = Completely agree). Higher scores correspond to more thorough information processing (α = 0.839; ω = 0.842).

*Perceived threat to freedom (as used in [70]).* This scale evaluated the extent to which participants feel that the messages are coercive, manipulative, or overly persuasive with four items (e.g., “The messages I have received have tried to restrict my freedom of choice”) rated on a 7-point Likert scale (1 = Completely disagree; 7 = Completely agree). Higher scores correspond to a greater perceived threat (α = 0.845; ω = 0.862).

*Emotions triggered by the messages (as used in [69]).* This scale measured the emotions triggered by the messages. Participants rated the extent to which they experienced each of 15 emotions when reading the messages on a 7-point Likert scale (1 = Not at all; 7 = Extremely). Of these, three items related to anger (annoyed, irritated, bothered; α = 0.832; ω = 0.837); three items related to fear (afraid, frightened, intimidated; α = 0.822; ω = 0.823); three items related to anxiety (agitated, worried, restless; α = 0.745; ω = 0.754); three items related to hope (hopeful, optimistic, encouraged; α = 0.788; ω = 0.793); and three items related to contentment (serene, calm, peaceful; α = 0.912; ω = 0.916).

### 2.6. Analyses

All analyses were conducted in R Studio (R version 4.4.2; [71]). All statistical analyses were conducted using an alpha level of 0.05 to determine significance.

For each scale, we calculated descriptive statistics at the item level, including mean, standard deviation, skewness, and kurtosis. Items were considered normally distributed if skewness was below |2| and kurtosis was below |7| [72]. We then examined the correlations between items within each measurement scale, expecting moderate-to-high correlations (*r* > 0.20) for items representing the same latent variable [73]. To assess the validity of the scale, we conducted an Exploratory Factor Analysis (EFA) for the scales that were specifically adapted or developed for this study (i.e., food safety practices, awareness, and self-efficacy). We used Parallel Analysis to determine the number of factors and evaluated factor loadings (acceptable above 0.35) and their uniqueness (acceptable if below 0.80). For the validated scales (i.e., message involvement, trust, systematic processing, perceived threat, and emotions), we conducted a Confirmatory Factor Analysis (CFA). The reliability of all scales was assessed using Cronbach’s alpha and McDonald’s Omega (acceptable threshold > 0.70 [72]), corrected item–total correlations (CITCs; *r* > 0.30; [74]), and “Alpha if Item is Deleted” (AiD) values. The total score of each scale was calculated as the average of the individual items.

In preliminary analyses, we ran descriptive and correlation analyses to examine the distribution of variables and their relationships. We tested for randomisation bias between conditions using one-way ANOVA and examined whether the message frame affected the frequency of message reading using a chi-square test. To examine differences in message evaluation (i.e., involvement, trust, systematic processing, perceived threat to freedom, and emotions) between the four message conditions, we used one-way ANOVAs.

To examine the effects of message valence congruence on food safety awareness (H1a, RQ1a), self-efficacy (H1b, RQ1b), and self-reported food safety practices (H1c, RQ1c), we conducted three mixed ANOVAs. Each analysis included one between-subject factor (message condition: control, PC, NC, PI, NI) and one within-subject factor (measurement time: Time 1 vs. Time 2). This approach was chosen to determine whether intervention effects varied across conditions and whether improvements persisted over time. Post hoc comparisons with Tukey’s correction were conducted where significant interaction or main effects were found to further explore group differences.

We then examined which message frame was most effective in increasing adherence to food safety practices based on participants’ initial levels of self-efficacy and food safety awareness (RQ2). This was performed using a moderation regression model, in which message condition was the independent variable, self-efficacy and food safety awareness at Time 1 were the moderators, and adherence to safe practices at Time 2 was the dependent variable. Moderators were centred by subtracting the mean from each value [75]. For significant interactions, simple slope analyses were conducted to clarify the nature of the interactions [75].

**Figure 3:**
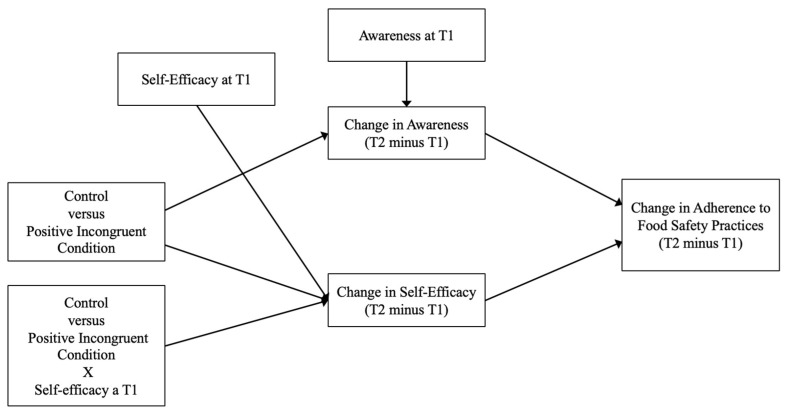
Theoretical moderated mediation model.

Finally, we examined the moderating role of food safety awareness and self-efficacy in the mediation pathway (RQ3; see Figure 3) through two separate moderated mediation analyses. These analyses used Hayes’ PROCESS macro (Model 7; [76]), with 5000 bootstrap samples to estimate the moderated mediation index. Here, message condition served as the independent variable, self-efficacy and food safety awareness at Time 1 as moderators, difference scores of self-efficacy and food safety awareness (Time 2 minus Time 1) served as mediators, and behaviour change (Time 2 minus Time 1) served as the dependent variable.

## 3. Results

Table 1 shows the descriptive statistics for the sociodemographic variables of the final sample. The sample had a balanced gender distribution, with most participants being unmarried (93%) and having a high school diploma (98%). Table 2 presents the mean and standard deviation of the key variables for the total sample and each condition. As shown in Table S1, none of the variables exhibited extreme skewness and kurtosis values, indicating that the assumption of normality was met. Items’ descriptive statistics, CICTs, and AiD values are presented in Table S2. The full psychometric evaluation of the scales, including detailed results, is available on OSF. Table 3 shows the correlations of the relevant variables, with the condition-specific correlations available on OSF.

To ensure comparability across conditions, we conducted one-way ANOVAs for the key variables at Time 1 (i.e., food safety awareness, self-efficacy, behaviour, and age across the five conditions). The results showed no significant effects of message condition on these variables (all *p* > 0.060; all *η_p_*^2^ < 0.01). Similarly, a chi-square test showed no significant gender differences across conditions, χ^2^(4) = 12.872, *p* = 0.378, Cramer’s *V* = 0.02, supporting the adequacy of randomisation.

### 3.1. Evaluation of the Messages

First, we determined the frequency with which participants read the messages in the experimental conditions (Table 1). On average, participants read 10.65 (SD = 1.63) messages during the intervention period, with no significant differences in reading frequency between message frames, *F*(3, 461) = 0.532, *p* = 0.661, *η_p_*^2^ < 0.01. Of the participants, 5.39% reported reading the messages rarely, 15.52% reported reading them often, and 79.09% reported reading them regularly. There were no differences in self-reported reading frequency between conditions, χ^2^(9) = 9.849, *p* = 0.363, Cramer’s V = 0.084.

We then used one-way ANOVAs to examine the differences in message evaluation and processing between the four conditions. The results showed that there were no differences in terms of message involvement (*F*(3, 460) = 0.504, *p* = 0.680, *η_p_*^2^ < 0.01), perceived tone (*F*(3, 460) = 1.365, *p* = 0.253, *η_p_*^2^ < 0.01), message trust (*F*(3, 460) = 0.673, *p* = 0.569, *η_p_*^2^ < 0.01), systematic processing (*F*(3, 460) = 0.145, *p* = 0.933, *η_p_*^2^ < 0.01), and perceived threat to freedom (*F*(3, 460) = 0.504, *p* = 0.680, *η_p_*^2^ < 0.01). In addition, there were no significant differences between conditions in perceived emotions, such as anger (Kruskal–Wallis test for violation of assumptions, χ^2^(3) = 7.123, *p* = 0.068, *η_p_*^2^ < 0.01), fear (*F*(3, 460) = 2.053, *p* = 0.106, *η_p_*^2^ = 0.013), anxiety (*F*(3, 460) = 1.973, *p* = 0.117, *η_p_*^2^ = 0.013), hope (Kruskal–Wallis test for violation of assumptions, χ^2^(3) = 0.885, *p* = 0.829, *η*^2^ < 0.01), and contentment (*F*(3, 460) = 0.727, *p* = 0.536, *η_p_*^2^ < 0.01), indicating that all messages were perceived equally across conditions in terms of involvement, trustworthiness, threat, systematic processing, and emotional impact. Table 2 shows the descriptive statistics for these ratings, and Table 3 shows their correlations.

### 3.2. Effects of the Messages on Food Safety Awareness, Self-Efficacy, and Compliance with Food Safety Practices

Regarding food safety awareness (H1a), the results showed a significant effect of time, *F*(1, 583) = 12.06; *p* < 0.001, *η_p_*^2^ = 0.007, indicating that the average level of awareness increases between Time 1 and Time 2 regardless of condition. This result could be due to a possible questionnaire effect. The mere participation in the study might have increased the participants’ awareness of the health consequences of proper or improper food preparation and storage. The main effect of condition, *F*(4, 583) = 3.37, *p* = 0.060, *η_p_*^2^ = 0.023 and the interaction between time and condition were not significant, *F*(4, 583) = 0.26; *p* = 0.900, *η_p_*^2^ = 0.002, suggesting that the average level of awareness increased significantly from Time 1 to Time 2, regardless of condition. Thus, H1a was not supported.

Regarding H1b, the results on self-efficacy showed a significant effect of time, *F*(1, 583) = 3.95; *p* < 0.047, *η_p_*^2^ = 0.007, indicating that the average level of self-efficacy increased between Time 1 and Time 2 regardless of condition. The effect of condition, *F*(4, 583) = 3.37, *p* = 0.010, *η_p_*^2^ = 0.023, was also significant. Finally, the results showed a non-significant interaction between condition and time, *F*(4, 583) = 1.17; *p* < 0.321, *η_p_*^2^ = 0.008. Thus, H1b was not supported.

Regarding compliance with food safety practices (H1c), the results showed a significant effect of time, *F*(1, 583) = 149.28; *p* < 0.001, *η_p_*^2^ = 0.204, and a non-significant effect of condition, *F*(4, 583) = 2.62, *p* = 0.265, *η_p_*^2^ = 0.009. In addition, the results showed a significant interaction between condition and time, *F*(4, 583) = 5.51; *p* < 0.001, *η_p_*^2^ = 0.036. Pairwise comparisons with Tukey’s correction showed a significant difference between the control (M = 3.69; SD = 0.55) and the *PC* (M = 3.91; SD = 0.58; *t*(583) = −3.07, *p* = 0.019, Cohen’s *d* = −0.386), the *NC* (M = 3.92; SD = 0.59; *t*(583) = −2.97, *p* = 0.026, Cohen’s *d* = −0.396), and the *NI* (M = 3.92; SD = 0.59; *t*(583) = −3.17, *p* = 0.013, Cohen’s *d* = −0.396) conditions at Time 2. There were no significant differences between the control and *PI* conditions (M = 3.87; SD = 0.62; *t*(583) = −2.33, *p* = 0.369, Cohen’s *d* = −0.316) at Time 2. Overall, these results partially confirmed our hypothesis and suggested that while compliance with food safety practices generally improved over time, the effectiveness of message conditions varied. Specifically, the *PC*, *NC*, and *NI* messages were significantly more effective than the control condition in improving food safety practices at Time 2. In contrast, the *PI* condition was not significantly different from the control condition, suggesting that not all message conditions were equally effective. These results underscore the importance of the message content for food safety behaviour.

### 3.3. Moderation of Self-Efficacy and Food Safety Awareness on Message Effectiveness

The overall model fit revealed a statistically significant effect, *F*(14, 573) = 2.91, *p* < 0.001, R^2^ = 0.066). The R^2^ value indicated that 6.6% of the variance in the dependent variable was significantly explained by the predictors. The coefficients of the direct effects of condition were positive and significant (*p* < 0.005; see Table 4), while the coefficients for the moderators were not significant, with p values above 0.05. As for the interaction terms between self-efficacy and the message conditions, the results showed a significant positive interaction with the *PI* condition (β = 0.097, SE = 0.0466, *t* = 2.0827, *p* = 0.038), suggesting that as self-efficacy increases, the positive effect of these messages on behaviour change becomes stronger. A simple slope analysis revealed that the effectiveness of these messages was significant for medium (β = 0.18, *t* = 2.79, *p* = 0.010) and high (β = 0.31, *t* = 3.55, *p* < 0.001) self-efficacy but not at low self-efficacy (β = 0.04, *t* = 0.45, *p* = 0.650).

As for the moderation effect of food safety awareness, the results showed a significant negative interaction between the *PI* condition and the moderator (β = −0.176, SE = 0.0790, *t* = −2.2270, *p* = 0.026), suggesting that the positive effect of these messages on behaviour change weakens as awareness increases. A simple slope analysis revealed that the effectiveness of these messages was significant for low (β = 0.320, *t* = 3.53, *p* < 0.001) and medium (β = 0.18, *t* = 2.79, *p* = 0.010) levels of awareness but not for high ones (β = 0.040, *t* = 0.44, *p* = 0.660). This suggests that individuals with lower levels of awareness are more receptive to messages emphasising food safety benefits and avoidance recommendations. In addition, the negative interaction between the *PC* condition and awareness was marginally significant (β = −0.132, SE = 0.078, *t* = −1.698, *p* = 0.090), indicating a possible moderating effect that should be investigated further.

### 3.4. Moderated Mediation

Regarding the moderating role of self-efficacy (Table 5, top panel), the analysis revealed that only the *PI* condition significantly predicted post-intervention levels of self-efficacy (β = 0.366, SE = 0.164, *t* = 2.237, *p* = 0.026). In addition, the mediator was a significant predictor of behavioural change (β = 0.041, SE = 0.013, *t* = 3.192, *p* = 0.002). All message conditions showed significant direct effects on behaviour change (all *p* < 0.013). The interactions between the message conditions and the moderator (self-efficacy at Time 1) were significant for the congruent conditions, i.e., *NC* (β = −0.545, SE = 0.111, *t* = −4.908, *p* < 0.001) and *PC* (β = −0.233, SE = 0.104, *t* = −2.244, *p* = 0.025), suggesting that for individuals in these conditions, the strength of the effect of the messages on post-intervention self-efficacy varied over pre-intervention levels of self-efficacy.

The simple slope analysis showed that the *NC* condition was effective for individuals with low (β = 1.002, SE = 0.2082, *p* = 0.026) and high (β = −0.527, SE = 0.236, *p* = 0.026) levels of pre-intervention self-efficacy but not for individuals with intermediate levels (β = 0.238, SE = 0.1587, *p* = 0.135). The direction of the effects suggests that for individuals with lower pre-intervention self-efficacy, exposure to messages with only negative content increases their self-efficacy. Conversely, the same message has a negative effect on post-intervention self-efficacy scores for those with higher pre-intervention self-efficacy. A simple slope analysis for the *PC* condition revealed that it had a positive effect on people’s post-intervention self-efficacy if they had a low level of self-efficacy before the intervention (β = 0.604, SE = 0.201, *t* = 3.010, *p* = 0.003) and not if they had medium (β = 0.277, SE = 0.149, *t* = 1.859, *p* = 0.064) and high (β = −0.049, SE = 0.216, *t* = −0.228, *p* = 0.820) levels of self-efficacy before the intervention. However, examination of the moderated mediation index for the two conditions (Table 6, top panel) revealed that the conditional indirect effect of pre-intervention self-efficacy on the effect of the *PC* condition on behaviour change was not significant (index = −0.010, Boot 95% CI [−0.024, 0.001]), whereas it was significant for the *NC* condition (index = −0.023, Boot 95% CI [−0.043, −0.006]). More specifically, the indirect effect on pre-intervention self-efficacy was only significant for those who had low scores on this variable (β = 0.041, 95%CI [0.012, 0.079]) and not for those who had medium (β = 0.010, 95%CI [−0.004, 0.029]) and high (β = −0.022, 95%CI [−0.054, 0.003]) scores.

Regarding the moderating role of food safety awareness, the analysis revealed no direct effects of message conditions on post-intervention awareness levels (all *p* > 0.266; Table 5, bottom panel) and no interaction effects of pre-intervention awareness levels in this relationship (all *p* > 0.109). Post-intervention awareness significantly predicted behaviour change (β = 0.277, SE = 0.149, *t* = 1.859, *p* = 0.064). All bootstrapped 95% confidence intervals for the moderated mediation index included zero, indicating that there were no conditional effects of pre-intervention awareness on the mediation process (Table 6, bottom panel).

## 4. Discussion

The results of this study provide nuanced insights into how message framing, message congruence, and individual baseline characteristics influence food safety behaviours.

Consistent with previous meta-analyses [15,16,17,18], results showed that a 12-day message-based intervention was effective in improving food safety practices among young adults. However, the magnitude of change varied depending on message framing [39]. Specifically, we found that participants exposed to emotionally consistent messages (i.e., PC and NC conditions) showed the greatest improvement in self-reported food safety behaviours, confirming previous research on the effectiveness of congruent emotional framing to improve message clarity and persuasiveness [47,48,49]. These findings suggest that message coherence achieved through emotion-focused cues (positive or negative) enhances the effect on preventive behaviours by reducing cognitive dissonance and improving message clarity.

Our results also show that incongruent message framing can be effective under certain conditions. Specifically, combining negatively framed belief-based content with positively framed skill-based instructions (NI condition) significantly improved food safety practices. Therefore, negatively framed beliefs combined with positively framed skills can still promote desired behaviours. This is consistent with findings from dual-process models such as the Extended Parallel Process Model [77], which states that fear appeals are most effective when followed by efficacy-enhancing messages. This negative–positive sequence is likely to increase attention and perceived threat before the cognitive dissonance is resolved by actionable, positively framed instructions, thereby maintaining engagement without overwhelming the audience [51,52]. In contrast, the ineffectiveness of positive incongruent messages (positively worded beliefs combined with negatively worded skills) can be attributed to a mismatch in motivational cues. While positively framed beliefs emphasise safety and benefits, they may not create the urgency needed to complement the avoidance-oriented actions recommended in the negatively framed skills. This discrepancy likely makes the recommended behaviour seem less relevant or compelling, reducing the persuasiveness and coherence of the message overall. Consequently, the inconsistent emotional appeal between the belief and skill components may explain the lower effectiveness of positive incongruent messages in promoting compliance with food safety practices.

While previous food safety interventions have demonstrated their effectiveness in improving food safety knowledge and awareness (although many studies use uncontrolled before-and-after studies rather than rigorous RCT designs [16,17,18]), our results showed a significant increase in food safety awareness in all conditions, including the control group. This suggests that mere exposure to the questionnaire at Time 1, particularly due to the repeated thematization of food safety issues, may have contributed to increased awareness among participants in the control group. This pattern is consistent with previous research showing that questionnaires themselves can influence awareness [78,79,80], particularly when the format encourages reflection on the topic. To avoid this potential influence, future research should consider methods that minimise the influence of questionnaire exposure on measures of awareness. However, it is noteworthy that this questionnaire-induced effect did not affect behaviour, as no significant increase in food safety practices was observed in the control group. This difference underscores that while awareness can be sensitive to repeated exposure and self-reflection, actual behaviour change requires more targeted communication interventions. These findings highlight the need to go beyond awareness alone and use strategically framed messages to achieve food safety behaviour change.

Previous research showed that educational programs that include lessons, quizzes, and discussions [31] or intensive, hands-on training combined with continuous reinforcement [32] significantly increase the self-efficacy of school canteen workers. In contrast, our study did not find a significant increase in self-efficacy in the experimental conditions compared to the control group. This discrepancy may be attributed to the shorter duration and lower intensity of the messaging intervention, which, unlike the face-to-face training, did not include any interactive components or personal feedback. This difference emphasises the importance of providing clear, actionable guidance that empowers individuals, especially when using digital communication strategies. Although the results were not significant, a descriptive review of the results showed a trend towards greater self-efficacy in all experimental groups, while the control group showed a decline after the intervention. This pattern suggests that while participants in the control group gained greater awareness of the health implications of safe practices, the lack of actionable guidance may have inadvertently reduced their sense of self-efficacy (e.g., “I now understand the importance of food safety, but I am unsure of what practices to use”). These findings highlight the need not only to raise awareness but also to provide clear, actionable strategies that enable individuals to put knowledge into practice. This is consistent with previous health behaviour research that emphasises the role of self-efficacy in promoting behaviour change [37]. Although the current findings are inconclusive, they underscore the importance of integrating practical skills training into food safety interventions to effectively enhance self-efficacy and promote sustained behaviour change. Future research should further investigate this dynamic to provide a more solid empirical foundation.

Beyond the influence of message framing alone, our results emphasise the role of individual characteristics of the receiver in moderating the effectiveness of message framing, which is consistent with Regulatory Fit Theory [21]. The results showed that pre-intervention self-efficacy had a significant impact on how participants responded to the intervention, especially in the positive incongruent messages (PI condition). In this condition, the combination of positively framed beliefs and negatively framed skills was particularly effective for those with medium-to-high self-efficacy but had little effect on those with low self-efficacy. This finding is consistent with previous research [60,63,81] showing that avoidance-oriented messages are more effective for individuals who already have confidence in their abilities. This finding is also consistent with Self-Efficacy Theory [27], which states that individuals with greater confidence in their abilities are more likely to engage in and master challenging tasks. In this context, individuals with medium-to-high self-efficacy may have been better able to process the negatively worded skill-based recommendations and interpret them as constructive guidance to avoid errors rather than perceiving them as discouraging or overwhelming. The negatively framed skills likely provided these participants with a clear understanding of the potential pitfalls, allowing them to view the recommendations as actionable steps to avoid mistakes. Conversely, participants with low self-efficacy appeared to find the negatively framed skills more intimidating, which may have increased feelings of incompetence and decreased their motivation to apply the recommended practices.

In addition, we found that pre-intervention awareness of food safety practices significantly moderated the effectiveness of message framing. Individuals with low-to-medium baseline awareness gave stronger positive responses to positive incongruent messages (PI condition), whereas high awareness attenuated these effects. A similar, albeit non-significant, trend was observed for positive congruent messages (PC condition). Individuals with low baseline awareness of food safety practices may be at a pre-contemplation stage where they have not yet fully realised that they need to change their behaviour and are therefore less likely to be motivated by messages that emphasise risks or negative consequences [82]. Instead, they may respond better to messages that emphasise the positive outcomes of change, as these are perceived as more motivating and less threatening. However, they may still prefer advice on what to avoid, as such advice, even without full risk awareness, offers concrete and actionable steps that reduce uncertainty and provide clear guidance on behaviours to avoid, facilitating a first step towards incremental change.

Finally, we found that self-efficacy significantly moderated the mediating pathway, especially for negative congruent messages (NC condition). Individuals with initially low self-efficacy showed a significant improvement in their self-efficacy after the intervention, leading to positive changes in their food safety behaviours. This finding suggests that for individuals with low baseline self-efficacy, exposure to emotionally consistent messages may improve perceived competence and ability, which are critical for the adoption of preventive health behaviours. In contrast, awareness did not significantly moderate the mediation pathway, suggesting that baseline awareness levels had no effect on the relationship between changes in awareness and adherence to safe food practices. This finding suggests that awareness may act as a background factor that favours an increase in general knowledge but does not directly influence the pathway from intervention to behaviour change. This underscores the notion that while awareness is necessary for informed decision-making, perceptions of self-efficacy ultimately drive behavioural adoption, supporting previous findings [26,28].

### 4.1. Limitations

Despite the contribution of the study, some limitations should be considered. First, the use of self-reports of adherence to food safety practices may lead to social desirability bias, limiting the accuracy of reported behaviour change. While future studies should consider incorporating objective measures to provide a more accurate assessment of compliance with food safety practices, it remains a challenge to obtain truly objective measures in this area. Food safety behaviours often take place in private settings, making direct observation or reliable monitoring methods difficult to implement without influencing the behaviour itself. Second, the duration of the intervention was relatively short at 12 days, so long-term behavioural changes may not be captured. Extending the intervention period and conducting follow-up assessments could help determine whether the observed improvements in food safety practices persist over time. In addition, it is possible that some types of messages may have a more lasting impact or require more time to have significant effects, as individuals may need more time to internalise the information and develop new habits. Third, the sample consisted primarily of unmarried young adults who had completed high school. Future research should test the intervention with different demographic groups, particularly older adults or individuals with different educational backgrounds, to examine whether the effects of the intervention differ in different subgroups. A final limitation of this study is the recruitment method, which was based on psychology students inviting participants through their personal networks. This may have led to a selection bias such that the sample was skewed towards younger and more motivated individuals. Future studies should use randomised recruitment strategies to increase external validity.

### 4.2. Study Implications

This study extends the theoretical understanding of message framing, congruence, and individual baseline characteristics in influencing health behaviour change. In particular, our findings emphasise the need to consider the Stages of Change Model [82] as a framework for tailoring health communication strategies. The differential impact of congruent and incongruent messages suggests that individuals who are at different stages of behavioural readiness will respond to different communication strategies. For example, individuals who are in the pre-contemplation or contemplation stage may benefit more from positively framed messages, whereas individuals who are closer to action may require directive or risk-oriented communication. Future research should further explore how the stages of change influence the receptivity and effectiveness of message framing, contributing to a dynamic and tailored approach to health communication. This aligns with recent findings on the stages of change in food-related decisions [83,84,85] and supports the development of stage-specific interventions that effectively guide people through the continuum of behaviour change.

Furthermore, our results contribute to Self-Efficacy Theory [27] by showing that self-efficacy moderates cognitive processing of message framing. This suggests that self-efficacy influences not only behaviour but also how individuals interpret and absorb the content of the message. Future research could investigate the cognitive mechanisms underlying these effects to create a more comprehensive theoretical model of health communication.

This study also challenges traditional framing effects [39] by showing that incongruent messages can be effective under certain conditions, suggesting that emotional congruence is not generally superior. This introduces the concept of Tailored Incongruence, which states that the strategic use of incongruent messages can increase engagement and behaviour change when tailored to individual psychological profiles.

Overall, these theoretical contributions underscore the importance of tailoring health communication to individual psychological profiles, leading to a more nuanced and dynamic model of health behaviour change communication.

In terms of practical implications, this study highlights the potential of carefully designed message-based interventions to improve young adults’ food safety practices. This study highlights innovative ways to improve food safety behaviours through targeted, psychologically based communication strategies. Beyond standard recommendations, results point to the potential of precision health communication that leverages individual psychological profiles to optimise intervention outcomes. First, the integration of dynamic adaptation mechanisms into mHealth applications could significantly improve their effectiveness. By continuously assessing users’ self-efficacy and awareness of food safety using interactive features, interventions could dynamically adjust the framing and content of messages in real time.

Second, the study highlights the value of message valence congruence but also opens up the possibility of exploring the strategic use of mixed valence. Specifically, combining negatively worded belief-based content with positively worded skill-based instructions can balance threat appraisal with actionable solutions, mitigating defensive responses while maintaining engagement. Especially in contexts characterised by behavioural inertia, where people resist change due to ingrained habits or perceived difficulties in adopting new behaviours, tailored interventions are critical. For example, young adults who habitually neglect safe food handling may continue to do so because these behaviours are more convenient or require less effort. In such cases, interventions should strategically break this inertia by highlighting both the risks of inaction (e.g., the potential for foodborne illness) and the ease of implementing safe behaviours, thereby reducing perceived barriers.

Third, the findings argue for a shift towards behavioural, integrated mHealth interventions that go beyond the provision of information. Incorporating gamification elements, progress tracking, and habit reinforcement techniques could transform static, message-based interventions into dynamic ecosystems that actively guide users through incremental behaviour change. These tools could embed food safety practices into daily routines, encouraging long-term adherence.

Finally, the study has implications for public health policy making. It suggests that food safety campaigns should leverage the scalability of digital platforms to disseminate tailored interventions at scale. Policy makers could use these insights to design national or regional initiatives that consider demographic and psychological variability, ensuring broader impact across diverse populations. Furthermore, integrating such digital interventions into educational systems could institutionalise safe food handling practices early, creating a generational ripple effect in public health outcomes.

These advanced strategies highlight the potential to merge psychological theory, digital innovation, and public health priorities, positioning food safety interventions as a model for precision health promotion in other domains.

## 5. Conclusions

This study contributes to the understanding of how message framing and individual psychological factors influence the effectiveness of message intervention. Although we observed an unexpected increase in food safety awareness that was due to exposure to the questionnaire rather than the intervention itself and only a descriptive trend that the intervention may have helped prevent a decline in self-efficacy over time, we found that message congruence improved self-reported adherence to food safety practices. Compliance also increased when people were exposed to negatively framed beliefs combined with positively framed skills. This supports the idea that fear-based messages are most effective when combined with clear, actionable solutions. In addition, we observed that high pre-intervention self-efficacy increased responsiveness to positive incongruent messages. This suggests that greater confidence in using food safety practices is associated with a preference for messages that recommend what should be avoided to prevent mistakes.

Future research should investigate long-term behavioural effects, different delivery methods, and dynamic mHealth interventions to further optimise food safety communication strategies. By integrating behavioural insights with digital health approaches, interventions can be refined to better target people and sustain positive food safety practices over time.

## Figures and Tables

**Table 1:** Sample descriptive statistics.

		Condition				
Variable	Overall	Control	PC	NC	PI	NI
	N = 588	*n* = 123	*n* = 130	*n* = 104	*n* = 97	*n* = 134
Gender						
Male	254 (43%)	47 (38%)	60 (46%)	40 (38%)	42 (43%)	65 (49%)
Female	332 (56%)	76 (62%)	70 (54%)	64 (62%)	54 (56%)	68 (51%)
Non-binary	1 (0.2%)	0 (0%)	0 (0%)	0 (0%)	0 (0%)	1 (0.7%)
Prefer not to answer	1 (0.2%)	0 (0%)	0 (0%)	0 (0%)	1 (1.0%)	0 (0%)
Mean Age (SD)	23.53 (3.68)	23.46 (3.67)	23.62 (3.89)	24.08 (3.47)	22.81 (3.13)	23.60 (3.95)
Educational Attainment						
Middle school diploma	3 (0.5%)	0 (0%)	0 (0%)	0 (0%)	1 (1.1%)	2 (1.5%)
High school, no diploma	9 (1.6%)	3 (2.5%)	3 (2.3%)	2 (1.9%)	0 (0%)	1 (0.8%)
High school diploma	145 (25%)	32 (27%)	41 (32%)	18 (17%)	28 (29%)	26 (20%)
Undergraduate student	154 (27%)	26 (22%)	35 (27%)	27 (26%)	25 (26%)	41 (31%)
Bachelor’s degree	199 (34%)	45 (38%)	38 (29%)	38 (37%)	32 (34%)	46 (35%)
Master’s degree	70 (12%)	14 (12%)	12 (9.3%)	19 (18%)	9 (9.5%)	16 (12%)
Missing	8	3	1	0	2	2
Marital Status						
Single	434 (79%)	96 (83%)	90 (76%)	71 (74%)	77 (85%)	100 (78%)
Cohabiting	76 (14%)	14 (12%)	14 (12%)	20 (21%)	7 (7.7%)	21 (16%)
Married	40 (7.3%)	5 (4.3%)	15 (13%)	5 (5.2%)	7 (7.7%)	8 (6.2%)
Prefer not to answer	38	8	11	8	6	5

Notes: PC = Positive congruent condition; NC = negative congruent condition; PI = positive incongruent condition; and NI = negative incongruent condition.

**Table 2:** Variables’ descriptive statistics (mean and standard deviation).

	Condition											
Variable	Control		PC		NC		PI		NI		Overall	
	*n* = 123		*n* = 130		*n* = 104		*n* = 97		*n* = 134		*n* = 588	
	T1	T2	T1	T2	T1	T2	T1	T2	T1	T2	T1	T2
Adherence to Food Safety Practices	3.62 (0.47)	3.69 (0.55)	3.61 (0.49)	3.91 (0.58)	3.63 (0.54)	3.92 (0.59)	3.63 (0.56)	3.87 (0.62)	3.64 (0.51)	3.92 (0.59)	3.63 (0.51)	3.86 (0.59)
Awareness	5.45 (0.79)	5.57 (0.77)	5.49 (0.72)	5.63 (0.82)	5.61 (0.75)	5.78 (0.87)	5.71 (0.81)	5.79 (0.85)	5.65 (0.86)	5.72 (0.88)	5.58 (0.79)	5.69 (0.84)
Self-Efficacy	6.96 (1.38)	6.88 (1.53)	7.16 (1.51)	7.33 (1.49)	6.99 (1.46)	7.24 (1.52)	7.45 (1.35)	7.63 (1.47)	7.23 (1.28)	7.26 (1.39)	7.15 (1.40)	7.25 (1.49)
Read Messages				10.62 (1.66)		10.53 (1.59)		10.81 (1.65)		10.63 (1.64)		10.65 (1.63)
Self-Reported Read Messages												
*Never*				0 (0%)		0 (0%)		0 (0%)		0 (0%)		0 (0.0%)
*Sometimes*				4 (3.1%)		6 (5.8%)		4 (4.2%)		4 (4.2%)		11 (8%)
*Often*				26 (20%)		10 (9.6%)		15 (16%)		15 (16%)		21 (16%)
*Always*				100 (77%)		88 (85%)		77 (80%)		77 (80%)		102 (76%)
Involvement				5.50 (1.02)		5.57 (0.96)		5.42 (1.04)		5.43 (1.09)		5.47 (1.03)
Trust				5.52 (0.92)		5.52 (0.84)		5.66 (0.86)		5.50 (0.86)		5.53 (0.87)
Systematic Processing				5.49 (0.87)		5.52 (0.86)		5.44 (0.88)		5.46 (0.91)		5.47 (0.88)
Perceived Threat				2.22 (1.09)		2.19 (1.02)		1.95 (0.92)		2.22 (1.05)		2.17 (1.05)
Perceived Anger				1.32 (0.50)		1.21 (0.37)		1.21 (0.45)		1.38 (0.63)		1.29 (0.51)
Perceived Fear				1.44 (0.60)		1.55 (0.63)		1.35 (0.55)		1.51 (0.67)		1.47 (0.62)
Perceived Anxiety				1.52 (0.58)		1.62 (0.61)		1.42 (0.52)		1.55 (0.64)		1.53 (0.59)
Perceived Hope				3.01 (0.79)		2.92 (0.90)		2.85 (1.00)		2.90 (0.82)		2.92 (0.87)
Perceived Contentment				3.49 (0.81)		3.43 (0.82)		3.49 (0.91)		3.35 (0.89)		3.44 (0.86)

Notes: PC = Positive congruent condition; NC = negative congruent condition; PI = positive incongruent condition; and NI = negative incongruent condition. T1 = Time 1; T2 = Time 2.

**Table 3:** Correlations between variables.

Variable	1	2	3	4	5	6	7	8	9	10	11	12	13	14
1. Adherence to Food Safety Practices T1														
2. Adherence to Food Safety Practices T2	0.65 **													
3. Awareness T1	0.19 **	0.11 **												
4. Awareness T2	0.19 **	0.31 **	0.50 **											
5. Self-Efficacy T1	0.23 **	0.21 **	0.21 **	0.18 **										
6. Self-Efficacy T2	0.27 **	0.34 **	0.16 **	0.26 **	0.59 **									
7. Involvement	0.21 **	0.33 **	0.09 *	0.24 **	0.08	0.16 **								
8. Trust	0.15 **	0.22 **	0.28 **	0.43 **	0.25 **	0.26 **	0.32 **							
9. Processing	0.25 **	0.42 **	0.19 **	0.38 **	0.21 **	0.19 **	0.63 **	0.41 **						
10. Threat	−0.05	−0.02	−0.08	−0.13 **	−0.10*	−0.14 **	−0.04	−0.20 **	−0.04					
11. Anger	−0.14 **	−0.20 **	−0.08	−0.16 **	−0.11*	−0.17 **	−0.31 **	−0.18 **	−0.22 **	0.28 **				
12. Fear	−0.12 *	−0.14 **	0.00	−0.01	−0.13 **	−0.20 **	−0.03	−0.08	0.03	0.23 **	0.38 **			
13. Anxiety	−0.11 *	−0.14 **	0.01	0.01	−0.12 **	−0.17 **	−0.00	−0.04	0.04	0.24 **	0.39 **	0.78 **		
14. Hope	0.21 **	0.32 **	0.07	0.22 **	0.12 **	0.09	0.43 **	0.33 **	0.44 **	0.00	−0.14 **	0.12 *	0.12 **	
15. Content	0.19 **	0.22 **	0.04	0.07	0.12 *	0.19 **	0.24 **	0.18 **	0.21 **	−0.05	−0.21 **	−0.26 **	−0.24 **	0.43 **

Notes: * indicates *p* < 0.05; ** indicates *p* < 0.01. T1 = Time 1; T2 = Time 2.

**Table 4:** Regression analysis results.

Predictor	β	SE	*t*	*p*
Intercept	0.061	0.042	1.452	0.147
PC Condition	0.230	0.059	3.937	<0.001
NC Condition	0.216	0.062	3.486	<0.001
PI Condition	0.179	0.064	2.790	0.005
NI Condition	0.224	0.058	3.867	<0.001
Self-Efficacy	−0.031	0.031	−1.028	0.304
Awareness	0.019	0.054	0.351	0.726
Self-efficacy x PC	0.063	0.041	1.528	0.127
Self-efficacy x NC	−0.009	0.044	−0.198	0.843
Self-efficacy x PI	0.097	0.047	2.083	0.038
Self-efficacy x NI	0.038	0.045	0.853	0.394
Awareness x PC	−0.132	0.078	−1.698	0.090
Awareness x NC	0.044	0.081	0.546	0.585
Awareness x PI	−0.176	0.079	−2.227	0.026
Awareness x NI	−0.059	0.072	−0.816	0.415

Notes: PC = Positive congruent condition; NC = negative congruent condition; PI = positive incongruent condition; and NI = negative incongruent condition.

**Table 5:** Results of moderated mediation analyses on self-efficacy (top panel) and awareness (bottom panel).

	**Δ Self-Efficacy (Mediator)**					**Behavioural Change (Dependent Variable)**				
**Path/Effect**	**β**	**SE**	***t***	***p***	**95% CI**	**β**	**SE**	***t***	***p***	**95% CI**
PC Condition	0.206	0.112	1.839	0.066	[−0.014, 0.426]	0.480	0.119	4.015	<0.001	[0.245, 0.318]
NC Condition	0.177	0.119	1.486	0.138	[−0.057, 0.410]	0.434	0.127	3.422	<0.001	[0.185, 0.683]
PI Condition	0.292	0.122	2.388	0.017	[0.052, 0.532]	0.363	0.129	2.812	0.005	[0.109, 0.616]
NI Condition	0.127	0.111	1.138	0.255	[−0.092, 0.345]	0.471	0.119	3.976	<0.001	[0.239, 0.704]
Self-efficacy T1	−0.167	0.082	−2.038	0.042	[−0.327, −0.006]					
PC x Self-efficacy T1	−0.266	0.109	−2.431	0.015	[−0.480, −0.051]					
NC x Self-efficacy T1	−0.605	0.117	−5.172	<0.001	[−0.834, −0.375]					
PI x Self-efficacy T1	−0.162	0.124	−1.300	0.194	[−0.406, 0.083]					
NI x Self-efficacy T1	−0.158	0.117	−1.351	0.177	[−0.389, 0.072]					
Δ Self-efficacy						0.041	0.038	3.192	0.017	[0.016, 0.067]
R^2^	0.231					0.109				
F	17.302			<0.001		11.881			<0.001	
	**Δ Awareness (mediator)**					**Behavioural Change (dependent variable)**				
**Path/Effect**	**β**	**SE**	***t***	***p***	**95% CI**	**β**	**SE**	***t***	***p***	**95% CI**
PC Condition	0.003	0.113	0.031	0.975	[−0.218, 0.225]	0.480	0.120	4.015	<0.001	[0.245, 0.318]
NC Condition	0.101	0.119	0.850	0.396	[−0.133, 0.335]	0.434	0.127	3.422	<0.001	[−0.185, 0.683]
PI Condition	0.066	0.122	0.543	0.588	[−0.173, 0.306]	0.363	0.129	2.812	0.005	[0.109, 0.616]
NI Condition	0.036	0.112	0.326	0.744	[−0.183, 0.256]	0.471	0.119	3.976	<0.001	[0.239, 0.704]
Awareness T1	−0.384	0.080	−4.776	<0.001	[−0.542, −0.226]					
PC x Awareness T1	−0.172	0.117	−1.455	0.146	[−0.402, 0.060]					
NC x Awareness T1	0.013	0.123	0.109	0.913	[−0.228, 0.255]					
PI x Awareness T1	−0.086	0.119	−0.723	0.471	[−0.321, 0.148]					
NI x Awareness T1	−0.092	0.107	−0.861	0.389	[−0.301, 0.118]					
Δ Awareness						0.240	0.040	6.064	<0.001	[0.162, 0.318]
R^2^	0.228					0.109				
F	17.082			<0.001		11.881			<0.001	

Notes: PC = Positive congruent condition; NC = negative congruent condition; PI = positive incongruent condition; and NI = negative incongruent condition.

**Table 6:** Conditional indirect effects of self-efficacy (upper panel) and awareness (lower panel).

Paths and Effects	β	Boot SE	Boot 95% CI
Condition → Self-efficacy → Behavioural Change			
PC Condition			
Low Self-efficacy T1	0.043	0.026	[0.002, 0.103]
Medium Self-efficacy T1	0.018	0.013	[−0.002, 0.050]
High Self-efficacy T1	−0.006	0.016	[−0.038, 0.030]
Index	−0.025	0.018	[−0.065, 0.003]
NC Condition			
Low Self-efficacy T1	0.071	0.036	[0.011, 0.150]
Medium Self-efficacy T1	0.013	0.016	[−0.011, 0.053]
High Self-efficacy T1	−0.041	0.027	[−0.098, 0.006]
Index	−0.058	0.028	[−0.117, −0.008]
PI Condition			
Low Self-efficacy T1	0.042	0.026	[0.002, 0.103]
Medium Self-efficacy T1	0.027	0.017	[0.001, 0.067]
High Self-efficacy T1	0.012	0.017	[−0.015, 0.053]
Index	−0.015	0.014	[−0.047, 0.009]
NI Condition			
Low Self-efficacy T1	0.026	0.018	[−0.001, 0.069]
Medium Self-efficacy T1	0.011	0.011	[−0.007, 0.037]
High Self-efficacy T1	−0.003	0.015	[−0.036, 0.028]
Index	−0.015	0.013	[−0.045, 0.004]
Condition → Awareness → Behavioural Change			
PC Condition			
Low Awareness T1	0.048	0.045	[−0.032, 0.152]
Medium Awareness T1	−0.004	0.025	[−0.052, 0.049]
High Awareness T1	−0.034	0.039	[−0.121, 0.034]
Index	−0.041	0.033	[−0.115, 0.017]
NC Condition			
Low Awareness T1	0.021	0.045	[−0.073, 0.107]
Medium Awareness T1	0.025	0.028	[−0.031, 0.077]
High Awareness T1	0.027	0.037	[−0.048, 0.100]
Index	0.003	0.029	[−0.052, 0.062]
PI Condition			
Low Awareness T1	0.040	0.053	[−0.060, 0.153]
Medium Awareness T1	0.014	0.028	[−0.04, 0.069]
High Awareness T1	−0.004	0.033	[−0.073, 0.058]
Index	−0.021	0.031	[−0.089, 0.037]
NI Condition			
Low Awareness T1	0.034	0.053	[−0.056, 0.126]
Medium Awareness T1	0.006	0.028	[−0.043, 0.056]
High Awareness T1	−0.012	0.033	[−0.079, 0.052]
Index	−0.022	0.028	[−0.081, 0.035]

Notes: PC = Positive congruent condition; NC = negative congruent condition; PI = positive incongruent condition; and NI = negative incongruent condition. T1 = Time 1.

## Data Availability

All materials and data presented in the study are openly available in OSF at https://osf.io/k8abj/.

## Supplementary Materials

The following supporting information can be downloaded at https://www.mdpi.com/article/10.3390/nu17050928/s1: Table S1: Variables’ descriptive statistics (mean, standard deviation, skewness, and kurtosis). Table S2. Items descriptive statistics (mean, standard deviation, skewness and kurtosis), Corrected Item Total Correlations, and Alpha if the Item is Dropped.
