# Supplementary material for: Communication Intervention to Improve Young Adults’ Food Safety Practices: The Benefits of Using Congruent Framing
Source: Nutrients. 2025 Mar 6;17(5):928. doi: 10.3390/nu17050928 (PMC11901861; doi:10.3390/nu17050928)
Supplement: Supplementary file 1 [file nutrients-17-00928-s001.zip › nutrients-3470741-supplementary.pdf]

nutrients

MDPI

Supplementary Materials

Communication Intervention to Improve Young Adults' Food Safety Practices: The Benefits of Using Congruent Framing

Table S1. Variables descriptive statistics (mean. standard deviation. skewness. and kurtosis).

Variable	Condition										Overall	
	Control N = 123		PC N = 130		NC N = 104		PI N = 97		NI N = 134		N = 588	
	T1	T2	T1	T2	T1	T2	T1	T2	T1	T2	T1	T2
**Adherence to Food Safety Practices**												
Mean (Sd)	3.62 (0.47)	3.69 (0.55)	3.61 (0.49)	3.91 (0.58)	3.63 (0.54)	3.92 (0.59)	3.63 (0.56)	3.87 (0.62)	3.64 (0.51)	3.92 (0.59)	3.63 (0.51)	3.86 (0.59)
Skewness	−0.41	−0.05	−0.13	−0.58	−0.31	−0.40	−0.06	−0.70	−0.08	−0.44	−0.18	−0.42
Kurtosis	3.09	2.34	3.16	3.25	2.56	2.57	2.63	3.27	2.35	2.99	2.79	2.86
**Awareness**												
Mean (Sd)	5.45 (0.79)	5.57 (0.77)	5.49 (0.72)	5.63 (0.82)	5.61 (0.75)	5.78 (0.87)	5.71 (0.81)	5.79 (0.85)	5.65 (0.86)	5.72 (0.88)	5.58 (0.79)	5.69 (0.84)
Skewness	−0.13	−0.31	−0.31	−0.51	−0.37	−0.65	−0.49	−0.77	−0.41	−0.55	−0.31	−0.53
Kurtosis	2.57	3.31	3.13	3.60	3.51	3.09	3.32	3.53	2.73	3.22	2.99	3.33
**Self–Efficacy**												
Mean (Sd)	6.96 (1.38)	6.88 (1.53)	7.16 (1.51)	7.33 (1.49)	6.99 (1.46)	7.24 (1.52)	7.45 (1.35)	7.63 (1.47)	7.23 (1.28)	7.26 (1.39)	7.15 (1.40)	7.25 (1.49)
Skewness	−0.40	−0.51	−0.76	−0.94	−0.69	−0.88	−0.18	−0.71	0.12	−0.01	−0.45	−0.61
Kurtosis	3.01	3.55	5.04	5.54	4.24	4.75	2.15	3.44	2.68	2.63	3.86	4.05
**Read Messages**												
Mean (Sd)				10.62 (1.66)		10.53 (1.59)		10.81 (1.65)		10.63 (1.64)		10.65 (1.63)
Skewness				−1.15		−0.84		−1.25		−1.19		−1.11
Kurtosis				3.37		2.58		3.49		3.56		2.56
**Involvement**												
Mean (Sd)				5.50 (1.02)		5.57 (0.96)		5.42 (1.04)		5.43 (1.09)		5.47 (1.03)
Skewness				−0.79		−1.27		−0.91		−0.95		−0.96
Kurtosis				3.56		6.71		4.30		4.41		4.55
**Trust**												
Mean (Sd)				5.52 (0.92)		5.52 (0.84)		5.66 (0.86)		5.50 (0.86)		5.53 (0.87)
Skewness				0.11		0.28		0.13		0.28		0.20
Kurtosis				2.04		2.23		2.12		2.23		2.21
**Systematic Processing**												
Mean (Sd)				5.49 (0.87)		5.52 (0.86)		5.44 (0.88)		5.46 (0.91)		5.47 (0.88)
Skewness				−1.47		−0.69		−0.68		−0.93		−0.99
Kurtosis				8.39		3.82		4.07		6.17		5.87
**Perceived Threat**												
Mean (Sd)				2.22 (1.09)		2.19 (1.02)		1.95 (0.92)		2.22 (1.05)		2.17 (1.05)
Skewness				1.43		1.68		0.95		0.99		1.36
Kurtosis				6.34		7.74		3.25		3.83		5.90

**Perceived Anger**					
Mean (Sd)	1.32 (0.50)	1.21 (0.37)	1.21 (0.45)	1.38 (0.63)	1.29 (0.51)
Skewness	2.11	2.11	3.05	2.07	2.44
Kurtosis	8.02	7.81	14.02	7.45	9.97
**Perceived Fear**					
Mean (Sd)	1.44 (0.60)	1.55 (0.63)	1.35 (0.55)	1.51 (0.67)	1.47 (0.62)
Skewness	1.68	1.04	1.62	1.39	1.45
Kurtosis	5.80	3.46	4.92	4.50	4.75
**Perceived Anxiety**					
Mean (Sd)	1.52 (0.58)	1.62 (0.61)	1.42 (0.52)	1.55 (0.64)	1.53 (0.59)
Skewness	1.42	0.82	1.33	1.23	1.23
Kurtosis	5.39	2.88	4.42	4.15	4.29
**Perceived Hope**					
Mean (Sd)	3.01 (0.79)	2.92 (0.90)	2.85 (1.00)	2.90 (0.82)	2.92 (0.87)
Skewness	−0.09	−0.07	−0.32	−0.58	−0.31
Kurtosis	3.05	2.67	2.24	2.81	2.80
**Perceived Contentment**					
Mean (Sd)	3.49 (0.81)	3.43 (0.82)	3.49 (0.91)	3.35 (0.89)	3.44 (0.86)
Skewness	−0.54	−0.01	−0.53	−0.50	−0.43
Kurtosis	3.87	2.40	3.48	3.31	3.38

Notes. PC = Positive Congruent Condition; NC = Negative Congruent Condition; PI = Positive Incongruent Condition; NI = Negative Incongruent Condition.

Table S2. Items descriptive statistics (mean, standard deviation, skewness and kurtosis), Corrected Item Total Correlations, and Alpha if the Item is Dropped.

			T1						T2					
**Construct**	**Item**	**Wording**	**Mean**	**SD**	**Skew**	**Kurtosis**	**CICT**	**AID**	**Mean**	**SD**	**Skew**	**Kurtosis**	**CICT**	**AID**
**Adherence to food safety practices**	1	I use pans in excellent condition to cook food.	4.37	0.72	−1.23	2.31	0.27	0.72	4.34	0.79	−1.44	2.77	0.39	0.80
	2	I check the inside of the meat to determine if it is well−cooked.	4.16	1.00	−1.30	1.33	0.26	0.73	4.27	0.99	−1.60	2.38	0.33	0.80
	3	I disinfect sponges and dishcloths at least once a week.	3.36	1.27	−0.23	−1.02	0.43	0.70	3.52	1.29	−0.53	−0.77	0.54	0.78
	4	I clean work surfaces with a detergent after finishing meal preparation.	4.19	0.96	−1.07	0.46	0.39	0.70	4.16	0.98	−1.22	1.18	0.50	0.79
	5	I wash my hands with soap before preparing a meal.	4.64	0.71	−2.31	5.78			4.69	0.63	−2.28	5.71		
	6	I wash my hands with soap when I start preparing a different type of food.	3.27	1.29	−0.16	−1.10	0.49	0.69	3.71	1.15	−0.57	−0.55	0.58	0.78
	7	I wash my hands with soap after meal preparation.	4.11	1.05	−0.98	0.01	0.43	0.70	4.32	0.90	−1.33	1.30	0.50	0.79
	8	I wash the cutting board when I start preparing a different type of food.	3.50	1.17	−0.39	−0.69	0.50	0.69	3.79	1.14	−0.72	−0.29	0.57	0.78
	9	I use clean utensils or wash those I have used when I start preparing a different type of food.	3.79	1.16	−0.60	−0.67	0.45	0.70	4.06	1.02	−0.95	0.16	0.55	0.79

	10	When I defrost frozen food. I place it in the refrigerator or use the microwave.	3.55	1.21	−0.59	−0.54	0.24	0.73	3.67	1.31	−0.76	−0.54	0.26	0.81
	11	I cook defrosted food within 24 hours.	4.70	0.61	−2.34	6.54			4.62	0.75	−2.58	7.85		
	12	I keep water cool and away from heat sources.	4.46	0.80	−1.61	2.75			4.60	0.76	−2.28	5.80		
	13	To store running water. I use bottles washed with soap.	2.78	1.58	0.12	−1.55	0.28	0.73	3.21	1.57	−0.28	−1.45	0.25	0.81
	14	When I freeze food. I add a label with the freezing date and/or expiration date.	2.18	1.36	0.82	−0.65	0.36	0.71	2.83	1.53	0.10	−1.49	0.46	0.79
	15	I put food in the freezer only if it is not hot.	4.53	1.00	−2.46	5.45			4.49	1.05	−2.29	4.46		
	16	I avoid different foods coming into contact with each other during storage.	4.11	1.04	−1.22	1.00	0.32	0.71	4.38	0.87	−1.46	1.77	0.45	0.79
	17	I store food in well−sealed containers or bags.	4.56	0.69	−1.66	2.82	0.30	0.72	4.60	0.67	−1.77	3.23	0.42	0.80
	18	I clean the top of a food package (e.g.. box or can) before opening it.	2.19	1.28	0.81	−0.49	0.40	0.70	2.84	1.42	0.09	−1.31	0.47	0.79
	19	I store food leftovers in the refrigerator in a covered container.	4.26	1.14	−1.68	1.99	0.20	0.72	4.23	1.22	−1.64	1.58	0.24	0.81
	20	When storing leftovers. I separate raw and cooked foods by placing them in different containers.	4.31	1.12	−1.62	1.63			4.45	0.95	−2.06	4.09		
**Food safety awareness**	1	Incorrect food preparation is one of the causes of health problems.												
	2	Incorrect food storage is one of the causes of health problems.	5.70	0.99	−0.70	0.80	0.55	0.50	5.72	0.95	−0.89	2.24	0.70	0.68
	3	Having more information on proper food preparation and storage would contribute to health protection.	6.00	0.93	−1.16	2.92	0.43	0.64	6.01	0.94	−1.16	2.48	0.57	0.81
**Self−efficacy**	1	While watching television.	7.01	2.26	−0.77	0.08	0.55	0.90	6.98	2.25	−0.74	−0.04	0.61	0.92
	2	When you feel restless or bored.	6.48	2.22	−0.40	−0.36	0.64	0.89	6.62	2.16	−0.46	−0.20	0.71	0.91
	3	During holidays.	7.13	2.15	−0.70	0.20	0.53	0.90	7.30	2.05	−0.84	0.49	0.60	0.92
	4	When preparing meals for others.	8.55	1.69	−1.88	4.66	0.56	0.89	8.54	1.66	−1.98	5.29	0.63	0.92
	5	When you are angry or irritated.	6.24	2.13	−0.31	−0.35	0.71	0.89	6.45	2.14	−0.38	−0.28	0.75	0.91
	6	When you are very hungry.	6.95	2.09	−0.55	0.02	0.70	0.89	6.95	2.08	−0.62	0.17	0.77	0.91
	7	When you are sad.	6.62	2.19	−0.40	−0.29	0.66	0.89	6.69	2.22	−0.47	−0.32	0.71	0.91
	8	When you want to sit down and enjoy your food.	7.33	2.14	−0.77	0.22	0.65	0.89	7.45	2.01	−0.82	0.45	0.69	0.91
	9	When you have guests at home.	8.60	1.74	−1.75	3.58	0.52	0.90	8.62	1.66	−1.94	4.67	0.57	0.92
	10	When you have to prepare your own meals.	8.02	1.67	−1.07	1.86	0.66	0.89	7.99	1.76	−1.23	2.12	0.66	0.92
	11	When you have little time to dedicate to meal preparation.	6.09	1.97	−0.22	−0.15	0.65	0.89	6.42	2.05	−0.40	−0.10	0.69	0.91
	12	When you have to prepare many meals.	6.81	1.97	−0.47	0.14	0.63	0.89	7.06	1.94	−0.62	0.28	0.72	0.91
**Message involvement**	1	The messages I read... engaged me.							5.45	1.21	−1.11	1.86	0.66	0.80
	2	... stimulated reflection.							5.40	1.15	−0.92	1.24	0.74	0.72
	3	... were interesting.							5.58	1.21	−1.21	1.87	0.68	0.78
**Trust in the messages**	1	How much trust do you have in the information presented?							5.44	0.99	0.00	−0.58	0.77	0.91
	2	Do you believe the information presented is reliable?							5.56	0.94	0.12	−0.92	0.87	0.82
	3	Do you believe the information presented is truthful?							5.64	0.92	0.09	−0.95	0.81	0.87
**Systematic processing**	1	While reading the messages … I thought about which actions I could take based on what I read.							5.68	1.04	−1.20	2.95	0.66	0.80
	2	… I found myself making connections between the information and what I have read or heard elsewhere.							5.19	1.21	−0.80	0.91	0.61	0.82

	3	… I thought about how the information related to other things I know.	5.32	1.13	−0.81	1.16	0.62	0.81
	4	… I tried to think about the importance of the information for my daily life.	5.65	1.09	−1.26	3.02	0.70	0.79
	5	… I tried to relate the content of the information provided to my health.	5.55	1.14	−1.14	2.23	0.63	0.81
**Perceived threat to freedom**	1	The messages I read… tried to limit my freedom of choice.	2.09	1.14	1.36	2.21	0.66	0.81
	2	… wanted to influence my choices.	2.72	1.53	0.69	−0.53	0.62	0.85
	3	… tried to manipulate me.	1.83	1.06	1.83	4.12	0.79	0.77
	4	… tried to pressure me.	1.98	1.21	1.52	2.15	0.72	0.79
**Emotions triggered by the messages**	1	To what extent did reading the messages make you feel… annoyed?	1.29	0.57	2.09	4.68	0.76	0.77
	2	… irritated?	1.23	0.54	2.65	7.94	0.78	0.76
	3	… bothered?	1.33	0.66	2.46	7.43	0.76	0.78
	4	… scared?	1.48	0.73	1.50	1.75	0.68	0.75
	5	… frightened?	1.46	0.72	1.59	2.13	0.70	0.74
	6	… intimidated?	1.47	0.72	1.44	1.33	0.65	0.78
	7	… agitated?	1.38	0.64	1.68	2.56	0.64	0.60
	8	… worried?	1.76	0.84	0.89	0.04	0.69	0.56
	9	… uneasy?	1.44	0.69	1.66	2.84	0.66	0.58
	10	… hopeful?	2.62	1.07	−0.06	−0.69	0.74	0.60
	11	… encouraged?	3.10	1.04	−0.43	−0.29	0.74	0.60
	12	… optimistic?	3.05	1.00	−0.35	−0.19	0.65	0.69
	13	… calm?	3.32	0.92	−0.46	0.39	0.75	0.93
	14	… serene?	3.47	0.93	−0.36	0.11	0.88	0.82
	15	… peaceful?	3.53	0.94	−0.41	0.18	0.84	0.86
